# An estrogen-sensitive fibroblast population drives abdominal muscle fibrosis in an inguinal hernia mouse model

**DOI:** 10.1172/jci.insight.152011

**Published:** 2022-04-19

**Authors:** Tanvi Potluri, Matthew J. Taylor, Jonah J. Stulberg, Richard L. Lieber, Hong Zhao, Serdar E. Bulun

**Affiliations:** 1 Division of Reproductive Science in Medicine, Department of Obstetrics & Gynecology, and; 2 Department of Surgery, Feinberg School of Medicine, Northwestern University, Chicago, Illinois, USA.; 3 Department of Physical Medicine and Rehabilitation, Feinberg School of Medicine, Northwestern University, Chicago, Illinois, USA.; 4 Department of Biomedical Engineering, McCormick School of Engineering, Northwestern University, Evanston, Illinois, USA.; 5 Shirley Ryan AbilityLab, Chicago, Illinois, USA.

**Keywords:** Endocrinology, Muscle Biology, Fibrosis, Sex hormones, Skeletal muscle

## Abstract

Greater than 25% of all men develop an inguinal hernia in their lifetime, and more than 20 million inguinal hernia repair surgeries are performed worldwide each year. The mechanisms causing abdominal muscle weakness, the formation of inguinal hernias, or their recurrence are largely unknown. We previously reported that excessively produced estrogen in the lower abdominal muscles (LAMs) triggers extensive LAM fibrosis, leading to hernia formation in a transgenic male mouse model expressing the human aromatase gene (*Arom^hum^*). To understand the cellular basis of estrogen-driven muscle fibrosis, we performed single-cell RNA sequencing on LAM tissue from *Arom^hum^* and wild-type littermates. We found a fibroblast-like cell group composed of 6 clusters, 2 of which were validated for their enrichment in *Arom^hum^* LAM tissue. One of the potentially novel hernia-associated fibroblast clusters in *Arom^hum^* was enriched for the estrogen receptor-α gene (*Esr1*^hi^). *Esr1*^hi^ fibroblasts maximally expressed estrogen target genes and seemed to serve as the progenitors of another cluster expressing ECM-altering enzymes (*Mmp3*^hi^) and to upregulate expression of proinflammatory, profibrotic genes. The discovery of these 2 potentially novel and unique hernia-associated fibroblasts may lead to the development of novel treatments that can nonsurgically prevent or reverse inguinal hernias.

## Graphical abstract

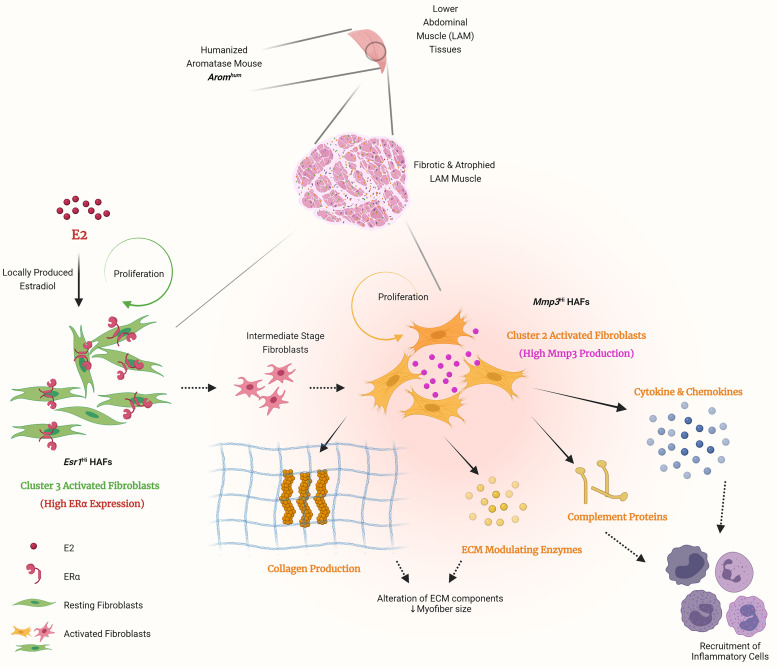

## Introduction

Inguinal hernias occur when abdominal viscera protrude through a weak area in the abdominal wall and can lead to intestinal obstruction. Inguinal hernias are extraordinarily prevalent, affecting up to 27% of men in their lifetime, and are the most common type of abdominal hernias (1, 2). Currently, the only treatment for a hernia is surgical repair, which carries the risk of several complications, particularly in populations with other preexisting conditions (3–5). Furthermore, hernia recurrence is difficult to manage surgically and leads to considerable disability and financial burden among patients. Despite the high morbidity and prevalence of hernias, the molecular mechanisms that drive hernia-associated muscular weakening remain unclear.

One potential causal factor in hernia development is changing levels of sex steroid hormones, which act on the abdominal wall muscles. The potent anabolic effects of androgens on skeletal muscles have long been documented, and abdominal muscles are no exception (6–8). For example, in one study, researchers found a strong positive correlation between serum testosterone levels and abdominal muscle area in young adult men (18–50 years) provided with exogenous testosterone (9). Via the enzyme aromatase, testosterone can be converted to estradiol, which also affects skeletal muscle. These effects were reported to be primarily protective and anabolic in peripheral (extremity-associated) skeletal muscles in both women and female mice (10–12), but little research on estrogen effects has focused on males or abdominal muscles. Notably, advanced age is associated with an increased risk of hernia. Circulating testosterone progressively declines, whereas estradiol and aromatase enzyme activity in peripheral tissues such as skeletal muscle and adipose tissue markedly increase with advancing age (13–19). Thus, a progressive decline in tissue testosterone/estradiol ratio may affect the integrity of abdominal muscles, particularly in older men, who are more prone to develop inguinal hernias.

Our laboratory generated the first mouse model of inguinal hernia through the expression of human aromatase (*Arom^hum^*) to provide insight into the molecular mechanisms that link sex steroid action, abdominal muscle weakness, and inguinal hernias (20, 21). In humans, aromatase converts androgens to estrogens in several extragonadal tissues, including skeletal muscle. Given that wild-type (WT) male mice express aromatase only in the brain, gonadal fat, and testes, our model mimics the human expression pattern by introducing a genomic fragment containing the entire human aromatase gene (*CYP19A1*) and its regulatory region, giving rise to its expression in other tissues such as skeletal muscle and fat as in humans (21). Male *Arom^hum^* mice show an aromatase-mediated increase in tissue estradiol levels and a decrease in circulating androgen and exhibit severe fibrosis and atrophy in lower abdominal muscle (LAM) tissue and consequently develop scrotal hernias (20). We further found that estrogen binding to the estrogen receptor-α (ERα) on LAM-resident fibroblasts promotes fibroblast proliferation and leads to fibrosis (20). Interestingly, other muscle groups (quadriceps, upper abdominal muscle [UAM] tissue) had lower fibroblast expression of ERα and were not affected by increased local estrogen levels (20). Estrogen-responsive genes (*Greb1* and *Pgr*) and profibrotic genes (*Spon2*, *Timp1*, and *Emb*) were exclusively upregulated in *Arom^hum^* LAM compared with *Arom^hum^* UAM or WT tissues; however, the cell types in LAM in which these transcriptomic changes occur remained unknown (20).

Multinucleated mature muscle fibers constitute the majority of skeletal muscle tissue by mass, but a diverse population of mononuclear cell types are resident in skeletal muscle. These include connective tissue fibroblasts that produce extracellular matrix (ECM), a mesenchymal progenitor cell type known as fibro-adipogenic progenitors (FAPs), immune cells, satellite stem cells, myoblast progenitor cells, endothelial cells, and glial cells. FAPs are marked by *Pdgfra* expression and are an intriguing muscle-resident fibroblast-like cell population (22). Previous work showed that in normal, injured, and fibrotic muscle, FAPs participate in crosstalk with other muscle cell types such as myoblasts (22, 23), satellite cells (24, 25), regulatory T cells (26), and macrophages (27). These signals are thought to contribute to myogenic differentiation and muscle repair/regeneration. However, the heterogeneity of FAPs, other ECM-producing muscle-resident fibroblasts, and their transcriptomic responses to hormone stimuli have been understudied.

To define the key cell types that respond to elevated local estrogen and initiate a fibrotic process leading to muscle weakness, we performed single-cell RNA sequencing (scRNA-Seq) on LAM tissues of WT and *Arom^hum^* mice. We produced what we believe is the first transcriptome map of abdominal wall muscle because previous muscular scRNA-Seq studies in mice focused exclusively on limb muscles or the diaphragm (28–32). We identified and validated the specific cell types that compose LAM and how these cell types and their transcriptome at the single-cell level differ between WT and *Arom^hum^* mice. This study provides insights into the cell map constituting LAM tissue and the effect of estrogen action on muscle-resident fibroblast gene expression. Our findings lay the groundwork for future studies exploring therapeutic targets to combat hernia formation and other fibrotic diseases associated with steroid hormone action in skeletal muscle.

## Results

### Single-cell RNA-sequencing atlas of LAM tissue cells from WT and Arom^hum^ mice.

**Figure 1:**
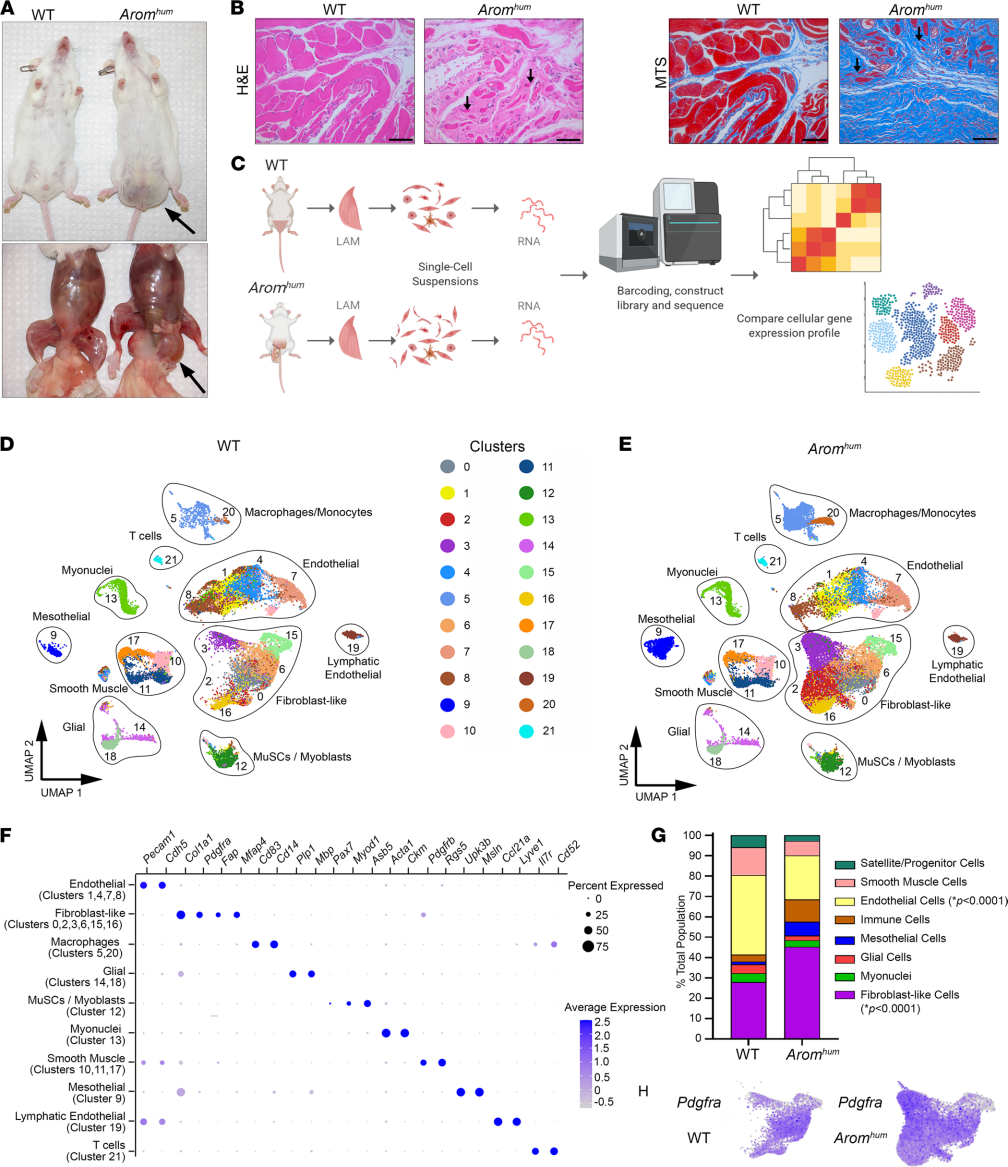
Identification of scRNA-Seq cell clusters through gene marker expression analysis in WT and *Arom^hum^* mice. (**A**) Representative images of male WT and *Arom^hum^* mice prior to (top) and after dissection (bottom). Arrows indicate bulging from scrotal hernias (*n* = 5). (**B**) Lower abdominal muscle (LAM) tissue sections from WT and *Arom^hum^* mice stained with hematoxylin and eosin (H&E, left) depicting marked reduction in myofiber size in *Arom^hum^* mice and Masson’s trichrome staining (MTS, right) indicating increased fibrotic deposition in LAM tissues of *Arom^hum^* mice. Arrows point to atrophied muscle fibers. Scale bar: 100 μm (*n* = 5). (**C**) Experimental design. LAM was harvested from 9- to 10-week-old male mice (*n* = 3 per group). Single-cell suspension was achieved through enzymatic digestion, and scRNA-Seq was performed using 10x Genomics Chromium. (**D**) UMAP plot of WT cells alone and (**E**) *Arom^hum^* cells alone. When both groups were analyzed together, 22 cell clusters were found, and cells were grouped into 10 cell groupings based on canonical marker expression. (**F**) Dot plot representing the expression of known marker genes of individual cell types. Size of dots corresponds to frequency of expression within a cell group. Color of dots corresponds to average expression level within the cell group. (**G**) Compositional makeup of the total cell population from WT and *Arom^hum^* mice, represented as a percentage of total cells. Groups from **D** and **E** were used, with macrophages/monocytes and T cells combined as “immune cells,” and the lymphatic endothelial cells combined with the other endothelial cell clusters. Data were compared using 2-way ANOVA and corrected for multiple comparisons. *P < 0.05. (**H**) UMAP feature plots showing expression of fibro-adipogenic progenitor (FAP) marker *Pdgfra* in a majority of fibroblast-like cells from LAM of WT and *Arom^hum^* mice (*n* = 3). MuSCs, muscle satellite cell/stem cells; UMAP, uniform manifold approximation and projection.

*Arom^hum^* male mice developed scrotal hernias starting from 4 weeks of age due to LAM tissue weakness. The contents within these hernias included abdominal viscera, gonads, gonadal fat, and urinary bladder (Figure 1A). H&E histology staining of LAM tissues revealed extensive muscle fiber atrophy only in *Arom^hum^* mice. Additionally, Masson’s trichrome staining showed widespread damage to LAM tissue by fibrosis in *Arom^hum^* mice (Figure 1B). However, we did not find apparent intramuscular adiposity as lipid accumulation was not observed by Oil Red O staining of LAM tissues from *Arom^hum^* mice (Supplemental Figure 1; supplemental material available online with this article; https://doi.org/10.1172/jci.insight.152011DS1). Together, these observations indicate an overall inability of LAM in *Arom^hum^* mice to contain the abdominal contents in place due to extensive fibrosis (20). To understand the transcriptional landscape and cellular makeup of LAM tissue in the context of fibrosis, atrophy, and elevated local estrogen action, we performed scRNA-Seq. LAM tissues were harvested from adult (9–10 weeks) male WT and *Arom^hum^* mice (*n* = 3 per group) manually and then enzymatically dissociated into a mononuclear single-cell suspension, from which cellular debris and red blood cells were removed. These single-cell suspensions were processed for scRNA-Seq on the 10x Genomics Chromium platform (Figure 1C).

Overall, we found 22 transcriptionally distinct clusters from an analysis of scRNA-Seq data from all WT and *Arom^hum^* mice combined (Figure 1, D and E). Some of these clusters represented similar cell types based on the top differentially expressed genes, illustrating intra–cell type heterogeneity in gene expression. We further identified 10 broader cell type groups based on canonical gene marker expression within the 22 clusters (Figure 1, D–F). The majority of cells were identified as either fibroblast-like cells (28% of total WT and 45% *Arom^hum^* cells) or endothelial cells (~40% and ~20%, respectively) (Figure 1G). Clusters 0, 2, 3, 6, 15, and 16 were designated as fibroblast-like cells due to their abundant expression of the FAP cell marker *Pdgfra* (Figure 1H) and genes encoding ECM-associated proteins such as *Col1a1* and *Mfap4*. Other known FAP cell markers (e.g., *Ly6a*, *Cxcl5*, *Ccl7*, *Dpp4*, *Tnc*, and *Wisp1*) were also expressed in fibroblast-like cells (Supplemental Figure 3). Endothelial cells were identified by their expression of known marker genes such as *Pecam1* and *Cdh5* and comprised 4 similar cell clusters (clusters 1, 4, 7, and 8). An additional cell cluster (cluster 19) within the UMAP was enriched for genes typically expressed in lymphatic endothelial cells, such as *Ccl21a* and *Lyve1*.

MuSCs and myoblast progenitors were also identified by scRNA-Seq analysis. Cluster 12 included genes suggestive of MuSCs and myoblast progenitor identities, such as the satellite cell and myoblast markers *Asb5* and *Myod1*. Furthermore, *Pax7*, the canonical satellite cell marker, and *Myf5*, a myogenic regulatory factor and satellite cell marker, were exclusively expressed in cluster 12 cells, leading us to conclude that this cluster was a mixed population of MuSCs and myoblasts (Figure 1, D–F). Cluster 13 expressed markers of mature myocytes, such as *Acta1* and *Ckm*, and the genes encoding myosin heavy chain proteins related to type I and type II skeletal muscle fibers, *Myh1*, *Myh2*, *Myh4*, and *Myh7*. Although our tissue processing method eliminated the large, multinucleated myofibers through size filtration, this cluster may represent a small number of myonuclei retained in the cell suspension as seen in previous muscle scRNA-Seq studies (30, 32). Other identified cell groups included macrophages/monocytes (clusters 5 and 20), mesothelial cells (cluster 9), smooth muscle cells (clusters 10, 11, and 17), glial cells (clusters 14 and 18), and T cells (cluster 21).

### Differences in LAM tissue cell type composition in WT and Arom^hum^ mice.

The WT and *Arom^hum^* mice displayed notable differences in the UMAP for specific cell types (compare Figure 1, D and E). Cell groups for WT and *Arom^hum^* mice were analyzed as percentages of the total number of cells per sample, with all immune cell types grouped together and lymphatic endothelial cells grouped with the other endothelial clusters (Figure 1G). There was a significantly larger proportion of fibroblast-like cells in *Arom^hum^* compared with WT in our sampling of LAM tissues (Figure 1, G and H, P = 0.0001). The percentage of endothelial cells was significantly lower in *Arom^hum^* than WT (Figure 1G). We also observed a lower, but not statistically significant, percentage of MuSCs/myoblasts and smooth muscle cells, with a parallel increase in mesothelial and immune cells in *Arom^hum^* than WT. When all individual clusters were analyzed, nonsignificant trends for a higher percentage of cells from *Arom^hum^* mice in specific fibroblast-like cell clusters (clusters 2 and 3) were observed (Supplemental Figure 3). Overall, fibroblast-like cells clustered into 6 populations, of which clusters 2 and 3 were more highly represented in the *Arom^hum^* mice (Figure 1, D, E, and H). These 2 fibroblast clusters contained the most striking difference between cells derived from WT and *Arom^hum^* tissue through UMAP visualization, which suggested a role in hernia development. We hypothesized that cluster 2 and 3 fibroblasts are linked to hernia causation and tentatively defined them as “hernia-associated fibroblasts” (HAFs).

### Esr1 expression in LAM tissue of WT and Arom^hum^ mice.

**Figure 2:**
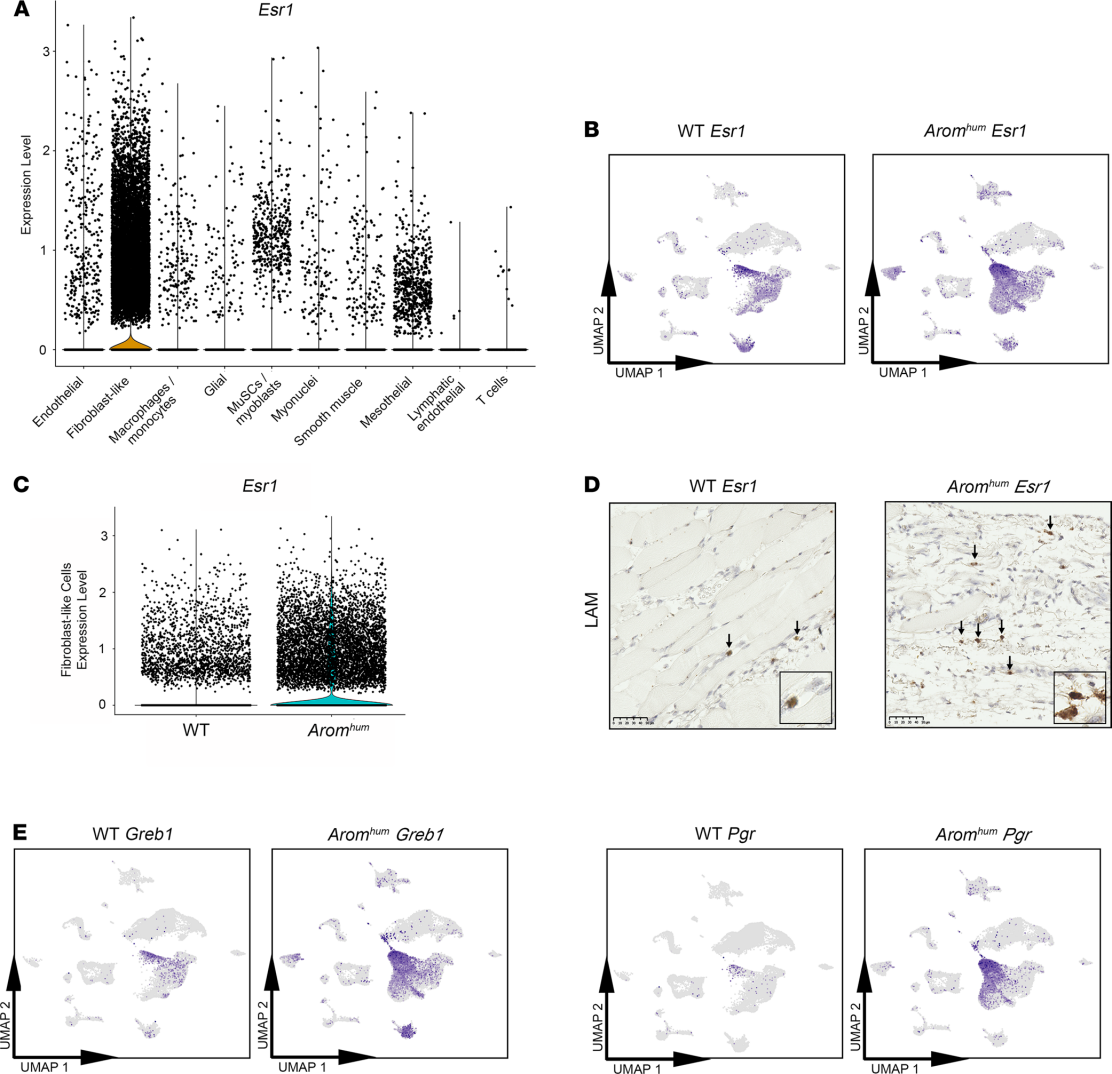
Expression of *Esr1* is predominantly in fibroblast-like cells of LAM tissue. (**A**) Violin plot of *Esr1* expression in 10 cell groupings from original UMAP of WT and *Arom^hum^* cells (Figure 1, D and E). Dots correspond to individual cells. (**B**) Feature plots representing *Esr1* expression in cells from WT mice and *Arom^hum^* mice on individual UMAP plots. Color intensity corresponds to relative expression. (**C**) Violin plot of *Esr1* expression in fibroblast-like cells of WT and *Arom^hum^* mice. (**D**) In situ hybridization (RNAscope) showing the expression of *Esr1* in the stromal region of WT and *Arom^hum^* LAM tissues (*n* = 5). Positive *Esr1* mRNA expression is demonstrated by brown, punctate staining as indicated by the black arrows. Scale bar: 50 μm. Insets: original magnification, 20×. (**E**) *Greb1* and *Pgr* (estrogen-responsive genes) feature plots for cells from WT mice and *Arom^hum^* mice on individual UMAPs. Color intensity corresponds to relative expression.

As expected, based on our previous work (20), the majority of LAM tissue cells that expressed *Esr1* (gene encoding ERα) were found in clusters within the fibroblast-like group of cells (Figure 2A). While *Esr1* expression was detected within all 6 cell clusters comprising the fibroblast-like cell group, it was highly enriched in the cluster 3 HAF population in both WT and *Arom^hum^*-derived cells (Figure 2, B and C). Expression of *Esr1* in WT and *Arom^hum^* LAM tissues was confirmed by RNAscope staining (Figure 2D). Higher *Esr1* expression was seen in *Arom^hum^* LAM compared with WT LAM, and the bulk of *Esr1* expression was noted in the fibroblast/stromal regions of muscle tissues, consistent with our scRNA-Seq results. Expression of known ERα target genes *Greb1* (33, 34) and *Pgr* displayed similar patterns of expression as *Esr1*, suggesting the presence of strong and active estrogen-mediated signaling, particularly in *Arom^hum^* LAM fibroblasts (Figure 2E).

### Transcriptomic differences between WT and Arom^hum^ fibroblast-like cells.

**Figure 3:**
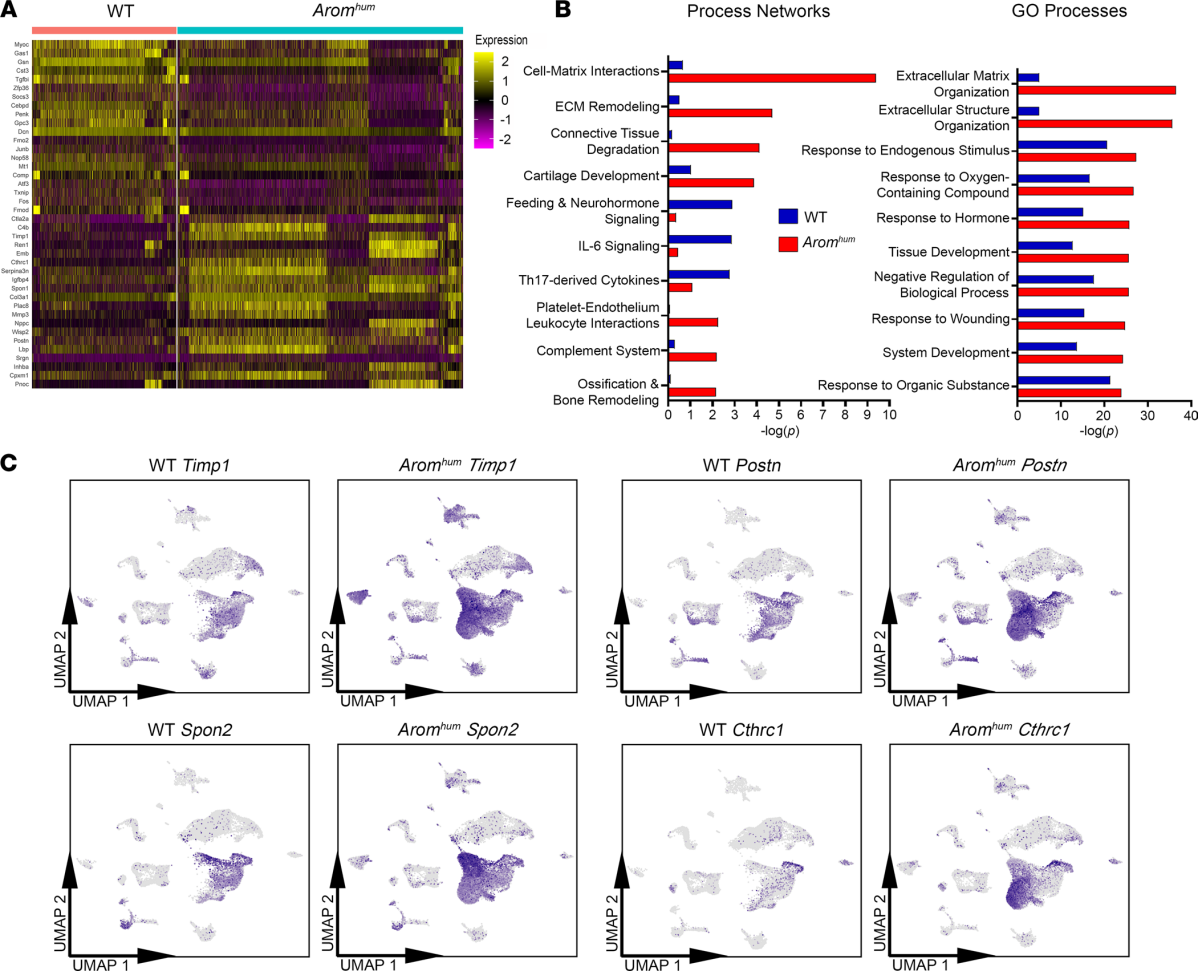
Transcriptional differences in fibroblast-like cells of LAM tissue between WT and *Arom^hum^* mice. (**A**) Heatmap of WT versus *Arom^hum^* mice for the fibroblast-like group of cells. The top 20 upregulated and top 20 downregulated genes for *Arom^hum^* mice are shown. (**B**) MetaCore analysis for process networks and Gene Ontology processes enriched in both WT and *Arom^hum^* fibroblast-like cells represented by –log(*P* value). (**C**) Feature plots of profibrotic genes *Timp1*, *Spon2*, *Postn*, and *Cthrc1* shown on individual UMAP plots for WT and *Arom^hum^* mice. Color intensity corresponds to relative expression.

*Arom^hum^* mice develop LAM tissue fibrosis, which arises partly from an expansion of fibroblasts in response to increasing local estrogen levels due to higher tissue aromatase activity (20). To further understand the mechanism of estrogenic action resulting in LAM tissue fibrosis, we explored the transcriptomic differences between WT and *Arom^hum^* fibroblast-like cells. Analysis of the top differentially expressed genes in WT and *Arom^hum^* mice highlighted enrichment of a fibrosis-related signature in *Arom^hum^* mice (*Timp1*, *Cthrc1*, *Spon1*, *Col3a1*, *Postn*, *Lbp*) (Figure 3, A and C). Pathway analysis in *Arom^hum^* mice revealed an enrichment of genes associated with cell adhesion, ECM organization and remodeling, connective tissue degradation, and response to hormone (Figure 3B). These processes and differentially expressed genes highlight the activation of a fibrotic/tissue remodeling state in fibroblast-like cells in *Arom^hum^* LAM tissue. Genes involved in inflammation through the complement system were also enriched in *Arom^hum^* LAM fibroblast-like cells, mainly *C4b* gene expression (Figure 3A). However, genes that drive inflammation via IL-6 signaling and a Th17-derived cytokine immune response were downregulated in *Arom^hum^* compared with WT LAM fibroblast-like cells (Figure 3B). These data point to a Th2-like profibrotic environment in *Arom^hum^* LAM (35). Furthermore, ECM remodeling and other profibrotic genes such as *Timp1*, *Spon2*, *Postn*, and *Cthrc1* were most highly expressed in the HAFs (clusters 2 and 3) of the fibroblast-like cell populations (Figure 3C). Overall, there were substantial differences in gene expression between LAM fibroblast-like cells in WT versus *Arom^hum^*, with *Arom^hum^*-derived cells enriched for a profibrotic set of genes and pathways representing ECM organization and cell-matrix interactions. When visualized on the UMAP, many of these profibrotic gene differences were especially evident in the HAF population of cells.

### Fibroblast-like cell subpopulations in LAM tissue.

**Figure 4:**
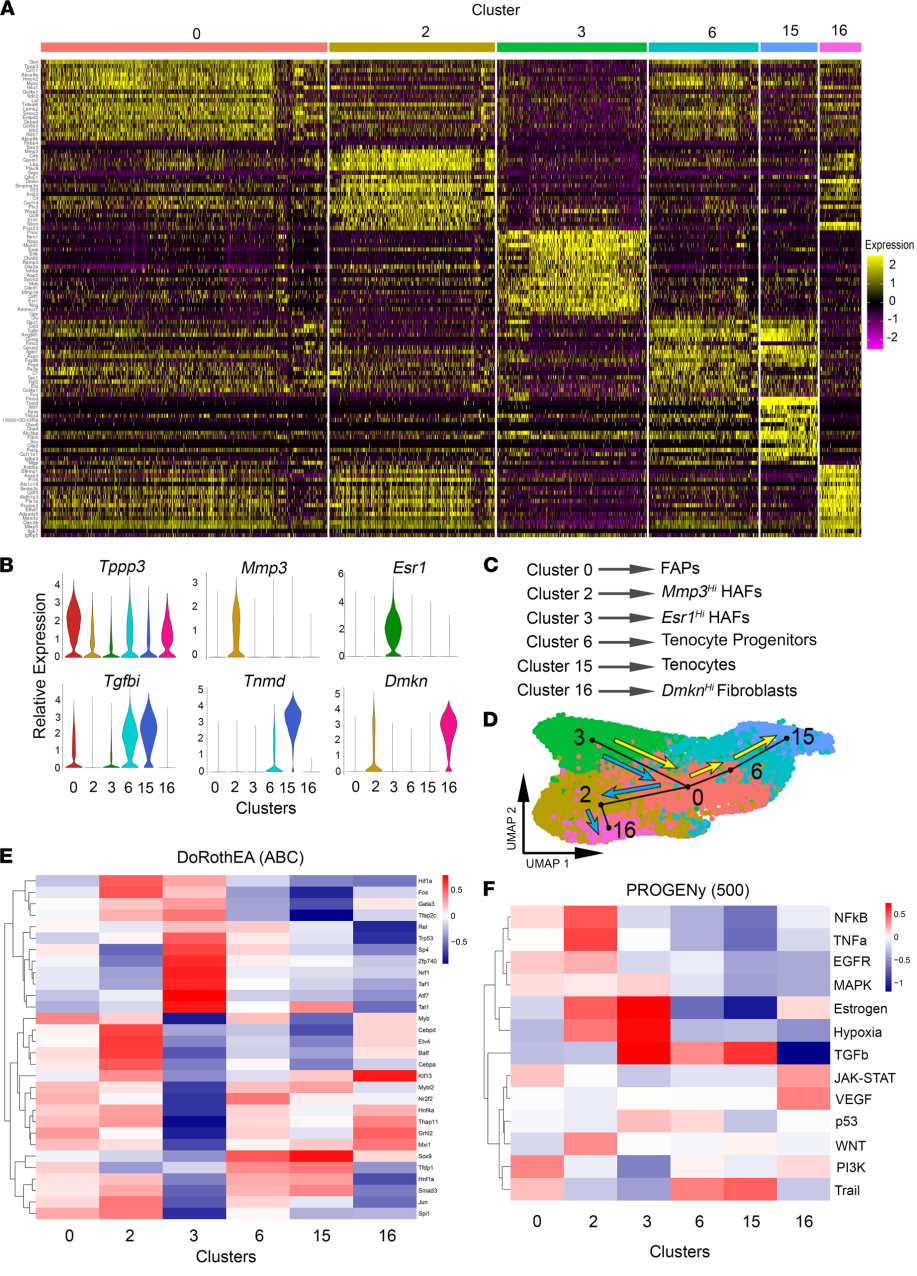
Six distinct clusters of fibroblast-like cells reside in LAM tissue of WT and *Arom^hum^* mice. (**A**) Heatmap of the top 20 differentially expressed genes in each of the 6 fibroblast-like cell clusters. (**B**) Violin plots of selected marker genes for the 6 clusters of fibroblast-like cells, showing relative gene expression in each cluster. (**C**) Summary of cell cluster identities based on differential gene expression. (**D**) Pseudotime analysis using the Slingshot package. UMAP of the only fibroblast-like cell group (from Figure 1, D and E) is shown. Start was forced at cluster 3 (*Esr1*^hi^ HAFs), and 2 potential lineages were found. One is denoted with blue arrows, the other with yellow arrows. (**E**) DoRothEA analysis on transcription factor activity within each of the 6 fibroblast-like cell clusters. (**F**) PROGENy analysis on pathway activity within each of the 6 fibroblast-like cell clusters.

**Figure 5:**
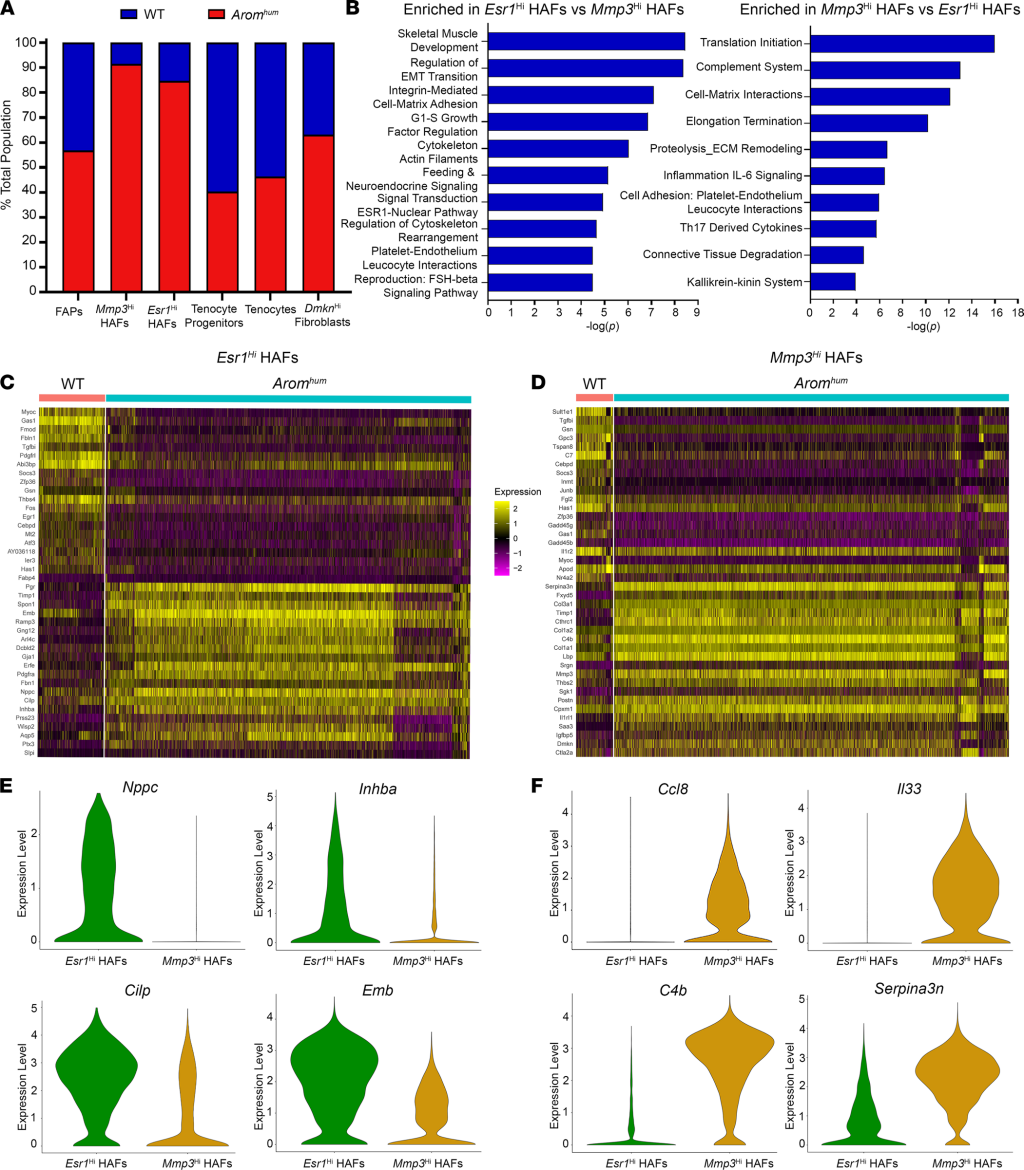
Two transcriptionally distinct clusters constitute HAFs. (**A**) Percentage contribution of WT (blue) and *Arom^hum^* (red) to the total cell count in each of the fibroblast-like cell clusters. (**B**) Top 10 process networks enriched in *Esr1*^hi^ HAFs compared with *Mmp3*^hi^ HAFs (left) and enriched in *Mmp3*^hi^ HAFs compared with *Esr1*^hi^ HAFs (right). (**C** and **D**) Heatmap of WT versus *Arom^hum^* for cluster 3 (*Esr1*^hi^ HAFs) and cluster 2 (*Mmp3*^hi^ HAFs). The top 20 upregulated and top 20 downregulated genes for *Arom^hum^* mice are shown in both. (**E**) Violin plots of selected genes highly expressed in the *Esr1*^hi^ cluster compared with the *Mmp3*^hi^ cluster. (**F**) Violin plots of selected genes highly expressed in the *Mmp3*^hi^ cluster compared with the *Esr1*^hi^ cluster.

To further understand the heterogeneity of fibroblast-like cells in LAM tissue, we analyzed transcriptomic differences between the 6 clusters of fibroblast-like cells described in Figure 1, D and E (clusters 0, 2, 3, 6, 15, and 16). The HAF populations of fibroblast-like cells, comprising clusters 2 and 3, had differentially expressed genes that distinguished them from each other and the other fibroblast-like cell clusters (Figure 4A). Cluster 2 contained high levels of *Mmp3*, a matrix metalloproteinase expressed in senescent fibroblasts that has a possible role in breast cancer progression (36), which led us to label this population as “*Mmp3*^hi^ HAFs” (Figure 4, A–C). This cluster also had a high expression of inflammatory and immune response–related markers, such as *Cxcl14*, *Lbp*, *C3*, *C4b*, *Saa3*, and *Il33*, and profibrotic markers *Timp1*, *Postn*, *Cthrc1*, and *Igfbp5* (Figure 4, A and B, and Supplemental Table 1). Cluster 2 also had several matrisome-related genes within its top 50 differentially expressed genes compared with other clusters, including *Col1a1*, *Col1a2*, *Col3a1*, and *Cpxm1* (Supplemental Table 1). We labeled cluster 3 as “*Esr1*^hi^ HAFs,” as the expression of *Esr1* and other estrogen-regulated genes (*Greb1*, *Pgr*, *Nppc*, *Cilp*, and *Rbp4*) was particularly high and specific to this population of cells (Figure 4, A–C, Figure 5E, and Supplemental Table 1). *Esr1*^hi^ HAFs also expressed high levels of profibrotic genes such as *Emb*, *Mfap4*, *Moxd1*, *Inhba*, and *Spon2* (Figure 4A and Supplemental Table 1). Cluster 0 contained the most cells of any fibroblast-like cluster and was nearly even in cell number in WT and *Arom^hum^* mice (Figure 4A and Supplemental Figure 1). While Cluster 0 lacked unique markers that differentiated it from the other fibroblast-like clusters, most cells in this cluster expressed FAP markers *Pdgfra* and *Ly6a*, which suggested that it also represented muscle-resident FAPs. These cells were also enriched for *Tppp3* (Figure 4, A and B), which has been suggested as a marker for tendon stem and progenitor cells (37). Therefore, we concluded that this cluster likely comprises a mixed population of multipotent FAPs, which may differentiate into tenocytes or fibroblasts.

Cluster 15 was denoted as tenocytes, as these cells expressed several tenocyte markers and related genes such as *Scx*, *Fmod*, *Tnmd*, *Comp*, *Wif1*, and *Thbs4* (Figure 4, A–C). Cluster 6 and cluster 15 expressed *Tgfbi*, *Angptl1*, *Prg4*, and *Cpxm2* (Figure 4, A and B). Cluster 6 also expressed low levels of some tenocyte markers, including *Tnmd* and *Fmod* (Figure 4, A and B). *Tppp3* was also expressed in 65% of cluster 6 cells, which suggested that these cells might be tenocyte progenitor cells (Figure 4B) (37). Finally, cluster 16 was a small cluster composed of nearly equal numbers of cells in WT and *Arom^hum^* mice. This cluster was marked by the expression of *Dmkn*, which encodes dermokine, first observed to be expressed in the epidermis. *Dmkn* has also been shown to be expressed in colorectal cancer (38) and pancreatic cancer and may be associated with epithelial-mesenchymal transition (39). Other keratinocyte-associated genes such as *Krtdap* and *Sbsn* were also expressed by cluster 16 (Figure 4, A and B).

To determine whether the 6 clusters of fibroblast-like cells had lineage relationships among one another, we performed pseudotime analysis using the Slingshot R package (40). Since *Esr1*^hi^ HAFs seemed to be the most estrogen-responsive cluster, we speculated that these cells are the original cell population that is activated in response to estrogen ligand. Therefore, to mimic the biological scenario of excess estrogen inducing fibroblast proliferation and differentiation, we started our lineage from cluster 3 *Esr1*^hi^ HAFs. From this analysis, we found 2 distinct lineages: i) cluster 3 → cluster 0 → cluster 2 → cluster 16 and ii) cluster 3 → cluster 0 → cluster 6 → cluster 15 (Figure 4D). Lineage 1 appears to be associated with hernia formation, whereas lineage 2 constitutes differentiation to mature tenocytes.

To predict computationally transcription factors associated with these 6 fibroblast clusters, we used the DoRothEA R package, which contains both human and mouse transcription factor regulons (41). *Esr1*^hi^ HAFs in cluster 3 showed especially high activity for the transcription factors *Trp53*, *Sp4*, *Zpf740*, *Nrf1*, *Taf1*, *Atf7*, and *Tal1*, while *Mmp3*^hi^ HAFs in cluster 2 had increased activity for *Hif1a*, *Fos*, *Cebpd*, *Etv4*, *Batf*, and *Cebpa* transcription factors (Figure 4E). Cluster 0 did not show high activity for any particular transcription factors, while *Sox9*, *Tfdp1*, *Hnf1a*, and *Smad3* were among the most active in clusters 6 and 15. Active transcription factors within cluster 16 included *Klf13*, *Hnf4a*, *Thap11*, *Grhl2*, and *Mxi1*. Additionally, we ran a Pathway RespOnsive GENes (PROGENy) analysis (42) to determine key pathways that were enriched in each fibroblast-like cell cluster. As expected, *Esr1*^hi^ HAFs in cluster 3 had high enrichment for estrogen signaling (Figure 4F). These cells showed high levels of hypoxia and TGF-β signaling as well. The *Mmp3*^hi^ HAFs in cluster 2 had a high activity of NF-κB and TNF-α signaling pathways, as well as moderately high levels of estrogen, hypoxia, and WNT signaling pathways. The most highly active pathway in cluster 0 FAPs was PI3K, and clusters 6 and 15 showed high activity for the TGF-β and Trail signaling pathways. Cluster 16 was enriched for genes from the JAK/STAT and VEGF pathways.

These data demonstrate that mesenchymal cells in the LAM of WT and *Arom^hum^* mice exhibit distinct heterogeneity, with specific genes and pathways enriched in each of the 6 clusters. It is possible that the increase in local estrogen in *Arom^hum^* LAM causes the expansion of the HAF populations (clusters 2 and 3), as well as differentiation from one HAF subtype to another, as proposed by pseudotime analysis (Figure 4D).

### Transcriptomic differences between the 2 LAM tissue HAF populations.

*Esr1*^hi^ and *Mmp3*^hi^ HAF clusters (clusters 3 and 2, respectively) both comprised the vast majority of cells from *Arom^hum^* samples (85% and 90%, respectively), whereas the other fibroblast-like cell clusters had a relatively even contribution of cells in WT and *Arom^hum^* LAM (Figure 5A). Upon discovering these potentially novel populations, we further characterized how they differed from one another and the differences between WT and *Arom^hum^* within each cluster. We performed pathway analysis on the differentially expressed genes in *Esr1*^hi^ HAFs relative to *Mmp3*^hi^ HAFs. As expected, one of the top process networks enriched in *Esr1*^hi^ HAFs was the Esr1-nuclear pathway (Figure 5B). Furthermore, G1-S growth factor regulation was upregulated in these cells, perhaps illustrating their proliferative capacity. Other enriched process networks included regulation of epithelial-mesenchymal transition, cytoskeleton-related pathways, and other reproduction-related pathways (Figure 5B). Interestingly, in *Mmp3*^hi^ HAFs, several processes related to inflammation and immune response were enriched compared with *Esr1*^hi^ HAFs (Figure 5B). Additional enriched pathways included ECM remodeling, connective tissue degradation, and translation-related processes (Figure 5B).

Within each HAF cluster, we also compared gene expression between WT and *Arom^hum^*-derived cells (Figure 5, C and D). These comparisons gave insights into genes in each HAF cluster that were affected by exposure to an estrogen-rich environment in *Arom^hum^* LAM tissue. Compared with *Esr1*^hi^ HAFs in WT LAM, the *Esr1*^hi^ HAFs in *Arom^hum^* LAM showed induction of estrogen-responsive genes such as *Pgr*, *Wisp2*, *Inhba*, *Cilp*, and *Nppc* as well as some fibrosis-associated genes such as *Timp1*, *Ptx3*, and *Aqp5* (Figure 5, C and E). Interestingly, *Esr1*^hi^ HAFs from *Arom^hum^* LAM also showed increased expression of the FAP marker *Pdgfra* (Figure 5C), suggesting an activated state compared with *Mmp3*^hi^ HAFs (43). *Mmp3*^hi^ HAFs derived from *Arom^hum^* LAM displayed an upregulation of inflammatory response genes including *Serpina3n*, *Fxyd5*, *C4b*, *Lbp*, *Ccl8*, *Il33*, *Il1rl1*, and *Saa3* (Figure 5, D and F). *Arom^hum^* *Mmp3*^hi^ HAFs also had higher expression of collagens (*Col1a1*, *Col1a2*, *Col3a1*) and other profibrotic genes (*Mmp3*, *Timp1*, *Cthrc1*, *Postn*, and *Igfbp5*) compared with WT *Mmp3*^hi^ HAFs (Figure 5D).

### Biological validation of HAF clusters in WT and Arom^hum^ LAM tissues.

**Figure 6:**
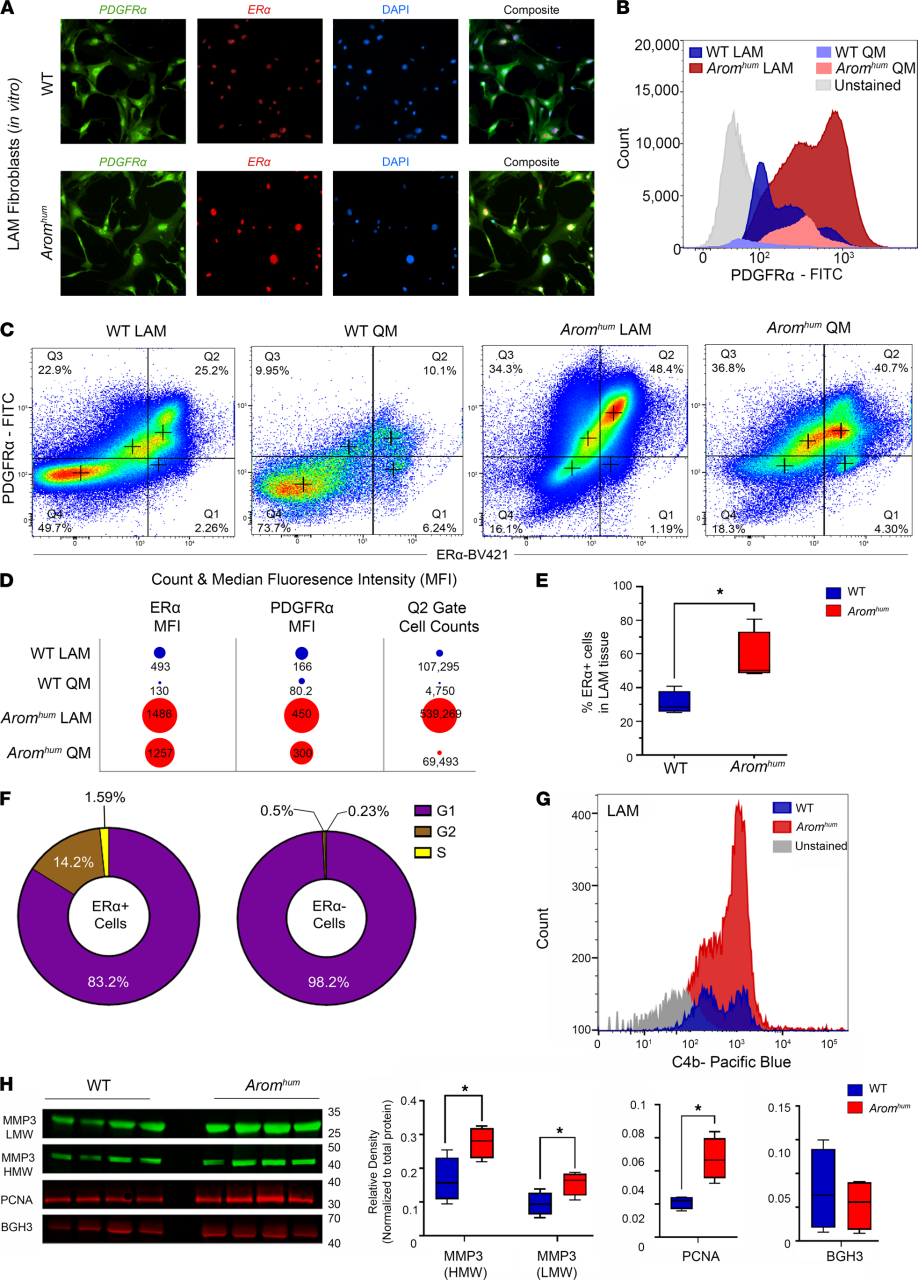
Validation of hernia-associated fibroblasts in WT and *Arom^hum^* LAM tissues. (**A**) Immunocytochemistry analysis of fibroblasts from LAM tissues of WT and *Arom^hum^* mice that were isolated via differential plating. PDGFRα and ERα expression were observed in both WT and *Arom^hum^* LAM fibroblasts (*n* = 10 mice/group in 4 technical replicates). (**B**) Flow cytometry histogram profiles of freshly isolated PDGFRα^+^ cells from LAM of WT and *Arom^hum^* mice. Quadriceps muscle (QM) was used as a control (*n* = 3). (**C**) Representative flow cytometry dot plots of ERα^+^ and PDGFRα^+^ cell populations (*n* = 3). (**D**) Dot plots representing the proportions of median fluorescence intensity (MFI) of ERα and PDGFRα and the number of cells in the Q2 gate of **C**. Numbers indicate MFI of ERα and PDGFRα and the cell counts for the plots in **C**. Both QM and LAM muscles were isolated uniformly from both WT and *Arom^hum^* mice (*n* = 3). (**E**) The total number of ERα^+^ cells from LAM tissues was quantified via flow cytometry in *Arom^hum^* versus WT mice (*n* = 20 mice/group, 5 technical replicates). (**F**) Cell cycle stages from freshly isolated LAM cells of *Arom^hum^* mice were analyzed via flow cytometry. DNA content of ERα^+^ and ERα^–^ cells was stained by FxCycle dye, and area histograms were used to quantify G1, S, and G2 phases (*n* = 5 mice/group). (**G**) Flow cytometry histogram profiles of complement protein C4b^+^ cells, indicating *Mmp3*^hi^ cluster, from LAM tissues of WT and *Arom^hum^* mice (*n* = 3). (**H**) Fluorescence Western blots of whole LAM tissue homogenates from WT and *Arom^hum^*. Two MMP3 bands were detected at ~45 kDa and ~27 kDa, PCNA protein was detected at ~30 kDa, and *Tgfbi* protein BGH3 was recognized at ~65 kDa. Quantification was performed by normalizing to total protein detected by Ponceau S staining (*n* = 4 mice/group). Box plots represent median with minimum and maximum values as whiskers and groups were compared using 2-sided *t* tests. *P < 0.05.

To validate the scRNA-Seq results, we first isolated all fibroblasts from WT and *Arom^hum^* LAM via differential plating technique (44). Immunocytochemistry staining showed that PDGFRα and ERα were present in these isolated fibroblasts (Figure 6A). Remarkably, all of these fibroblasts expressed both proteins, indicating only the activated *Esr1*^hi^ fibroblast populations were captured via this technique. To further validate and quantify *Esr1*^hi^ clusters in vivo, we performed flow cytometry in freshly isolated LAM cells, staining with antibodies against PDGFRα and/or ERα. We first observed substantially higher numbers of PDGFRα^+^ in *Arom^hum^* LAM compared with WT LAM (Figure 6B). We also compared these results to QMs from WT and *Arom^hum^* as controls. Both had fewer PDGFRα^+^ cells than LAM from the same mice. Furthermore, we found that *Arom^hum^* LAM had considerably higher proportions of the *Esr1*^hi^ cluster (PDGFRα^+^/ERα^+^) than WT LAM (48.4% vs. 25.2%, Figure 6C). Although *Arom^hum^* QMs had a similar proportion of PDGFRα^+^/ERα^+^ cells (40.7%), in the LAM tissue, the total number of PDGFRα^+^/ERα^+^ cells was greater, and their fluorescence intensity was substantially higher (Figure 6D). As expected, the percentage of ERα^+^ cells in LAM tissue was significantly higher in *Arom^hum^* than WT (*P* = 0.03, *n* = 20 mice/group, 5 technical replicates) (Figure 6E). To verify whether these *Esr1*^hi^ fibroblasts cells are the ones actively dividing in *Arom^hum^* LAM, we performed a flow cytometry analysis of DNA content in LAM fibroblasts staining with ERα. Approximately 16% of ERα^+^ cells were in S and G2 phases of cell cycle while less than 1% of ERα^–^ cells were in these phases, indicating active cell division primarily in ERα^+^ cells (Figure 6F, n = 5). In addition, cell proliferation marker PCNA was upregulated in *Arom^hum^* LAM, suggesting an overall increase in cell proliferation in these tissues (Figure 6H, n = 4, *P* = 0.04). Overall, these results indicate that *Arom^hum^* LAM fibrosis results from estrogen-induced *Esr1*^hi^ fibroblast proliferation.

To validate the *Mmp3*^hi^ cluster, we performed flow cytometry using an *Mmp3*^hi^ cell surface marker, complement protein C4b. Concurrent with the scRNA-Seq findings, we found a higher number of C4b^+^ cells in *Arom^hum^* LAM compared with WT LAM (Figure 6G). Furthermore, we performed Western blots to determine the levels of the MMP3 secreted protein in LAM tissue homogenates of WT and *Arom^hum^* mice. Two active and cleaved MMP3 proteins were expressed at significantly higher levels in *Arom^hum^* mice than WT mice (Figure 6H, n = 4, *P* = 0.049). Cluster 6 marker *Tgfbi* (BGH3) was similar between WT and *Arom^hum^* LAM tissues, as expected (Figure 6H).

## Discussion

Fibrosis represents a pathological response of tissue that affects various organs, including the liver (45), kidney (46), lung (47), heart (48), and skeletal muscle (49). There is no currently accepted treatment for pathological fibrosis, largely because the etiology is unknown. Using a potentially novel *Arom^hum^* mouse model of inducible hernia formation, we identified a population of modified and specialized fibroblasts in the LAM that we termed HAFs. We further demonstrate that, in addition to normal cells of mesenchymal origin found in WT LAM, *Arom^hum^* LAM contained 2 novel subpopulations within HAFs — one expressing genes indicative of estrogen responsiveness and the other expressing immune and inflammatory markers. As with many issues of nomenclature, we predict that HAFs are present and will be identified in other fibrosis models.

Our scRNA-Seq approach revealed what we believe is a previously undefined fibroblast-like cell heterogeneity, which clustered into 6 subpopulations based on transcriptomes. Because most cells in the fibroblast-like clusters (other than tenocytes, cluster 15) expressed the canonical FAP markers *Pdgfra* and *Ly6a* (which encodes Sca-1), it was difficult to determine which cells were FAPs versus differentiated fibroblasts, leading us to describe these cells as “fibroblast-like” (Supplemental Figure 3) (50–52). Consistent with previous scRNA-Seq studies in other muscle groups such as soleus and gastrocnemius, these fibroblast-like cells express genes such as *Cxcl5*, *Ccl7*, *Dpp4*, *Tnc*, *Wisp1*, *Cd34*, and *Dcn* (Supplemental Figure 3) (28, 29, 53). The differential gene expression of the clusters and pseudotime analysis suggested that cluster 0 are FAPs, as they seemed to be the most multipotent cluster of the fibroblast-like cell group. It is also possible that all clusters could be considered FAPs in different cell states or with varying degrees of differentiation, but our transcriptional analysis was unable to make this distinction (Supplemental Figure 2) (51).

We demonstrated that fibroblast-like cells expressed *Esr1*, which was increased in *Arom^hum^* fibroblast-like cells compared with WT, along with higher expression of ERα-associated genes. This estrogenic effect is likely due to the increased local estradiol in LAM tissue of *Arom^hum^* mice, which has high ERα expression (Figure 6E). As expected, within the fibroblast-like group as a whole, we saw an upregulation in fibrosis-related pathways (ECM remodeling/organization, cell-matrix interactions, and connective tissue degradation) in *Arom^hum^* compared with WT LAM. However, we were intrigued to find enrichment of the complement system — a prominent inflammatory response pathway — in *Arom^hum^* LAM fibroblast-like cells compared with WT (Figure 3B and Figure 6G). Estrogen has previously been linked to the complement system, where estradiol increased complement component C3 in rat uterine epithelial cells (54) and an estrogenic steroid upregulated C3 and complement factor B expression in the mouse endometrium (55). Estrogen may drive the activation of inflammatory pathways such as the complement system, which then contributes to the observed fibrosis phenotype. Inflammation and fibrosis have been widely described in the liver, kidney (56), lung (57), heart (58, 59), and skeletal muscle, and our findings suggest a potential causative mechanism, at least in the LAM. Notably, of the fibroblast-like cells, 2 clusters represent what we believe are previously uncharacterized HAF populations — *Esr1*^hi^ and *Mmp3*^hi^ — that appear to be hallmarks of estrogen-mediated hernia formation in *Arom^hum^* mice.

**Figure 7:**
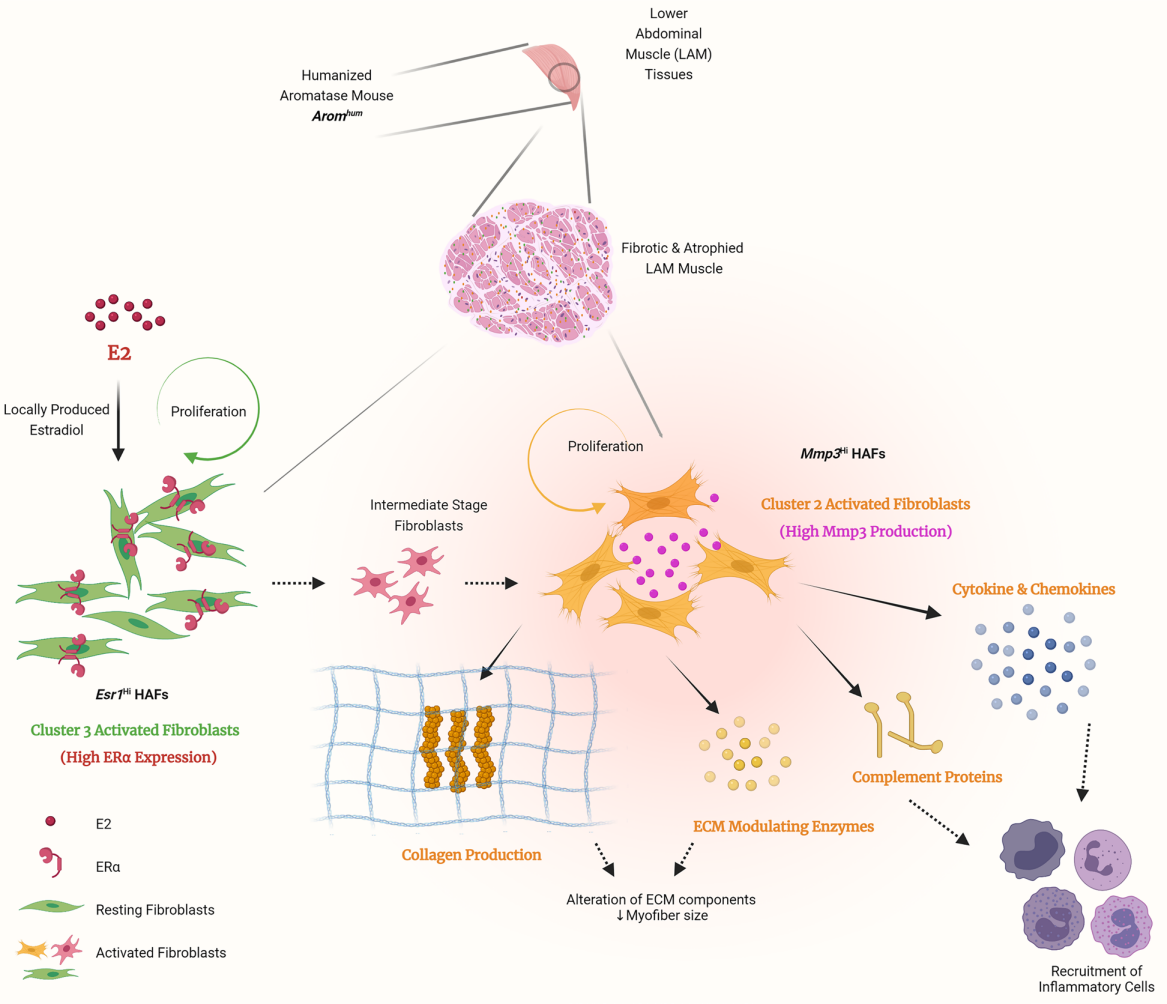
Schematic illustration of the proposed mechanisms of local estrogen-driven LAM fibrosis and inguinal hernia formation. Scrotal hernias in *Arom^hum^* mice are caused by weak lower abdominal muscle (LAM) tissues. Locally produced estradiol (E2) by aromatase in LAM activates *Esr1*^hi^ HAFs (green) to proliferate and presumably differentiate into *Mmp3*^hi^ HAFs (yellow) via an intermediate stage (red). *Mmp3*^hi^ HAFs synthesize elevated levels of collagens and ECM-modulating enzymes. These cells further produce complement proteins, cytokines, and chemokines that recruit immune cells, fostering a profibrotic, proinflammatory environment.

We previously identified estrogen as a possible causative factor in hernia formation in *Arom^hum^* mice, as it promoted proliferation of abdominal muscle-resident stromal cells specifically through activation of ERα (20). However, the factors activated by estrogen signaling that promote this proliferation are yet to be determined. The *Esr1*^hi^ HAFs (cluster 3) may represent activated FAPs due to estrogen signaling, as this cluster showed an abundance of *Pdgfra* expression as a whole, as well as an increase in the DNA content in S and G2 phases of the cell cycle in *Arom^hum^* compared with WT (Figure 5C and Figure 6, B–D) (43). By contrast, the *Mmp3*^hi^ HAF population (cluster 2) was enriched for several inflammatory and immune-related genes, indicating that this cluster has a different function than *Esr1*^hi^ HAFs. This cluster was also highly enriched for complement system–related genes compared with all fibroblast-like clusters (Figure 4A and Figure 6G) and specifically compared with *Esr1*^hi^ HAFs (Figure 7). Previous studies in arthritis have similarly pointed to a critical role of the complement system in priming the synovial fibroblasts into an activated state (60, 61). Another proinflammatory candidate, cytokine *Il33*, and its receptor *Il1rl1* are both upregulated in *Mmp3*^hi^ HAFs and are characteristic of fibrotic lung diseases, such as asthma (62). IL-33 is also a crucial regulator of inflammation in rheumatoid arthritis patients and mouse models alike, wherein it activates TNF pathways in fibroblasts (63, 64). Overall, the evidence points toward a Th2 cytokine–driven fibrosis in *Arom^hum^* LAM, modulated primarily by *Mmp3*^hi^ HAFs (35). Also of note, both HAF clusters showed enrichment for hypoxia signaling and *Hif1a* transcription factor activity (Figure 4, E and F). This is a significant finding since HIF-1α is a direct target of ERα in breast cancer cells (65), suggesting that estrogen-activated hypoxia signaling could also contribute to muscle fibrosis.

MMP3, also known as stromelysin-1, degrades several ECM components, such as collagens, laminins, and fibronectin, and has been known to exert oncogenic effects in prostate and breast cancers (66–68). We found an elevated expression of activated, cleaved MMP3 proteins in *Arom^hum^* LAM compared with WT (Figure 6H). Previous studies on fibroblasts isolated from patients with conjunctivochalasis and pterygium reveal a marked increase in MMP3 expression (69, 70). Elevated MMP3 is also associated with inflammatory arthritis, lupus, and mouse models of lung injury (71–75). Additionally, mice deficient in *Mmp3* are protected against lung injury with less influx of inflammatory cells and perivascular edema (76). MMPs are also distinctly upregulated in incisional hernias. Analysis of fascia from incisional hernia patients showed substantial elevations of MMP3 and MMP9, deregulation of ECM-altering enzymes, and alteration of collagen I/III ratios (77–79). Besides differences in circulating MMPs, tissue explants from inguinal hernia patients have been shown to produce higher MMP2, MMP9, TIMP1, and TIMP2 (80). Overall, our results of *Mmp3*^hi^ fibroblasts are comparable to previous research in incisional and inguinal hernia patients and inflammatory and autoimmune disease models.

Estrogen-mediated effects on skeletal muscle–resident FAPs and fibroblasts have not previously been studied to our knowledge. However, estrogen has previously been shown to promote proliferation and/or activation of tissue-specific fibroblasts, such as within uterine fibroids (81), gingival fibroblasts of periodontal connective tissue (82), dermal fibroblasts (83), periductal fibroblasts of the prostrate in neonatal rats (84), and rat cardiac fibroblasts (85). Estrogen effects on fibroblasts are especially evident in systemic sclerosis, where it has been demonstrated to increase fibroblast expression of fibronectin, collagen type I, and laminin (86, 87). Based on the results of our transgenic model, we propose that estrogen causes fibrosis through the proliferation of estrogen-responsive fibroblasts, upregulation of inflammatory pathways, and the resulting induction of tissue remodeling/profibrotic transcripts (Figure 6F and Figure 7). We further characterized the potential lineage relationships between these clusters through pseudotime analysis. This result provided a possible lineage of *Esr1*^hi^ HAFs differentiating into *Mmp3*^hi^ HAFs via a multipotent transitional FAP state (cluster 0) (Figure 4D).

Moreover, analyses such as pseudotime are only predictions based on gene expression of the 6 fibroblast-like clusters. Inferences made from such analyses must be confirmed through in vivo studies. To fully characterize the *Esr1*^hi^ HAFs, in vitro studies examining the effects of estrogen treatment on genome-wide gene transcription and extracellular matrix modifying proteins are necessary. Additionally, it would be interesting to study *Arom^hum^* LAM gene expression at earlier time points and after aromatase inhibitor or E2-ERα antagonist treatment to understand the mechanistic basis behind the early stages of fibrosis and atrophy. Additionally, the role of androgens or their deprivation in LAM HAFs from *Arom^hum^* mice is another important area of future research.

While this study focused on the heterogeneity and aromatase-driven shift in transcriptional programs of fibroblast-like cells within *Arom^hum^* LAM, this is only one of several cell types involved in the onset of hernias in this mouse model. Our previous work described severe myocyte atrophy as another hallmark of *Arom^hum^* mice (20). Due to the known anabolic actions of androgens, which stimulate muscle protein synthesis and build muscle mass through activation of androgen receptor (88), it is plausible that the aromatase-mediated decrease in circulating testosterone contributes to the observed atrophy phenotype in *Arom^hum^* mice. To adequately address whether changes in androgen and estrogen levels influence *Arom^hum^* myocyte atrophy, myofiber-related cells of all stages of myogenesis would need to be analyzed. Single-nucleus RNA sequencing (snRNA-Seq) has recently been employed to analyze the transcriptomes of mononuclear and multinucleated cells such as myofibers (89–92). Future work should apply snRNA-Seq to understand differences in transcriptional programs in WT and *Arom^hum^* mice for LAM MuSCs, myoblasts, and mature myonuclei and the effects of androgens and estrogens on these cells that lead to hernia formation.

In summary, we have identified potentially novel subpopulations of fibroblasts that participate in the fibrosis response and may contribute to formation of inguinal hernias. Specifically, we have identified 2 unique populations of HAFs, the *Esr1*^hi^ HAFs that appear to represent activated FAPs due to estrogen signaling and the *Mmp3*^hi^ HAF population that is enriched for several inflammatory and immune-related genes. This work demonstrates that the previously observed *Arom^hum^* stromal cell expansion and fibrosis phenotype is multifactorial and occurs through both the proliferation of existing fibroblasts and differentiation into pathogenic subtypes. This study paves the way to identify specific genes, proteins, and cell subtypes as therapeutic targets to treat fibrosis in a number of organ systems.

## Methods

### Mice.

Further information can be found in Supplemental Methods. All mice used for this study were 9- to 10-week-old male mice on an FVB/N background (Baylor College of Medicine Genetically Engineered Mouse Core, Houston, Texas, USA). The *Arom^hum^* mouse model was generated by our laboratory and has been previously described (20, 21). *Arom^hum^* transgenic mice express the human aromatase gene, including the promoter region, coding region, and 3′-polyadenylation site driven by its native tissue-specific promoters, resulting in a humanized tissue expression pattern of aromatase. Mice were maintained on a 14-hour light/10-hour dark cycle under the care of the Center for Comparative Medicine at Northwestern University. Standard chow (7912; Harland Tekland) and water were provided to mice ad libitum. Only male mice were used for the study as *Arom^hum^* female mice do not develop inguinal hernias. Male mice used for experiments were randomized and investigators were blinded to genotype group when measuring scrotum sizes. Mice with a scrotal area more than 145 mm^2^ were considered to have a hernia. All WT mice had scrotal area less than 140 mm^2^.

### Single-cell suspension.

LAM tissues from 3 WT mice and 3 *Arom^hum^* mice were harvested and processed on 3 separate dates, with tissue from 1 WT mouse and 1 *Arom^hum^* littermate mouse processed on each date. A previously published protocol for the isolation of mononucleated cells from limb skeletal muscle tissue (93) was modified. Each tissue was placed in cold wash media (Hyclone Ham’s F10 nutrient mixture with 1 mM l-glutamate [GE Life Sciences], 10% horse serum [Life Technologies], and penicillin-streptomycin [Omega Scientific]) until completion of tissue harvesting, then manually minced for about 5 minutes into a slurry. The tissue slurry was incubated in muscle dissociation buffer (wash media + 1000 U/mL collagenase II [Worthington Biochemical]) at 37°C with 70 rpm agitation for 1 hour. The cells were then washed with wash media and centrifuged at 500*g* for 5 minutes at 4°C. The supernatant was discarded with 4 mL of wash media remaining, and 500 μL of stock collagenase II (stock at 1000 U/mL) and 500 μL of stock Dispase II solution (stock at 11 U/mL) were added and incubated at 37°C with 70 rpm agitation for an additional 30 minutes. Cells were then passed through a 20-gauge syringe to further dissociate single cells, washed with wash media, and passed through a 40 μm filter to eliminate multinucleated cells. The cell suspension was then centrifuged at 500*g* for 5 minutes at 4°C, the supernatant was discarded, and red blood cell lysis buffer (BioLegend) was added per the manufacturer’s instructions. Cells were then counted with a hemocytometer and resuspended with an appropriate volume of 1× PBS with 1% BSA to achieve 1000–1500 cells/μL. The cells were finally passed through a 40 μm FLOWMI TIP cell strainer 2 times to remove any remaining cell debris and large clumps. The final cell suspension was kept on ice in DNA LoBind Tubes (Eppendorf) and submitted for scRNA-Seq.

### Single-cell RNA sequencing.

Samples for scRNA-Seq were examined first for cell viability, density, singularity, and prep quality, ensuring no clumps and cell debris. Samples passing the quality control process were loaded onto 10x Genomics Chromium to partition and encapsulate single cells into nanoliter-sized GEMs (Gel beads-in-EMulsion). A total of 6 samples were analyzed, with each targeting 10,000 cells. Single-cell suspension from LAM tissues with approximately 95% of cell viability was used as input material with an optimal cell concentration of 700–1200 cells/μL. In the Chromium, each encapsulated cell was lysed within its GEM. The total RNA released was reverse-transcribed to cDNA with primers attached to the gel bead, each of which carried a unique 10x Genomics barcode for downstream cell separation. Subsequently, the GEMs were broken, and all uniquely barcoded cDNAs were pooled and then amplified by PCR to generate enough material for Illumina sequencing. This library construction process for sequencing was carried out using the 10x Genomics Single-Cell 3′ Reagent Kit Protocol. Sequencing of the 10x Genomics libraries was performed on the Illumina HiSeq 4000, at a depth of approximately 30,000 reads per cell in the Northwestern University NUSeq Core.

### Data processing.

Transcripts were aligned to the mouse reference genome (mm10-3.0.0) with the human aromatase gene *CYP19A1* using Cell Ranger v3.0.2. The estimated number of cells per sample was 10,770 ± 853 on average, with 27,351 ± 2444 mean reads per cell. Of these reads, 92.1% were mapped to the reference genome on average. The median number of genes detected per cell was 2007 ± 123 in each sample.

### Single-cell RNA sequencing data analysis.

scRNA-Seq data analysis was conducted as previously described (94, 95). Quality control metrics were used as mentioned previously (94, 95). In brief, prior to analyzing the gene expression data, cells were filtered out by the feature count and gene number thresholds for quality control. Cells with <200 unique feature counts, >2500 feature counts, <1000 unique molecular identifiers, or >5% mitochondrial counts were removed from the analysis. Outliers were identified via median absolute deviation (MAD) and removed if they were more than 3 MADs from the median. Seurat v3 was used in R 4.0.0 for the data analysis. Data were first log normalized and scaled using *NormalizeData()* and *ScaleData()* functions (94, 96). Log normalization was performed with a scaling factor of 10,000 using the *NormalizeData()* function. All other Seurat functions were set to default parameters unless mentioned. Dimensionality reduction was performed using principal components analysis and UMAP. Dimensions for these analyses were estimated using Jackstraw and Elbow plots. Louvain algorithm was used to find clusters in Seurat. The function *FindAllMarkers()* was used to identify cluster markers (97). Cell types that displayed notable differences in gene expression (e.g., fibroblast-like cells) between WT and *Arom^hum^* mice were assigned to subsets and reclustered for further analysis. Differentially expressed genes were identified using *FindMarkers()* between 2 idents (e.g., WT vs. *Arom^hum^*) using Wilcoxon’s rank-sum test (96, 97). Pathway enrichment analysis was performed on differentially expressed genes using MetaCore (Clarivate Analytics). Pathway inference analysis on fibroblast clusters was performed using the PROGENy v1.12.0 R package (42, 98, 99). Pseudotime inference analysis of the fibroblast clusters was performed using Slingshot v1.8.0 Bioconductor R package, setting cluster 3 as the original cluster (40). To infer transcription factor activity, Virtual Inference of Protein-Activity by Enriched Regulon analysis (v1.24.0) along with mouse regulons from DoRothEA v1.2.0 were used (41, 98–100). Unless specified, arguments were set to default values.

### Fibroblast isolations and immunocytochemistry.

LAM tissues from 5 WT mice and 5 *Arom^hum^* mice were harvested and placed in cold wash media (Hyclone Ham’s F10 nutrient mixture with 1 mM l-glutamate [GE Life Sciences], 10% horse serum [Life Technologies], and penicillin-streptomycin [Gibco]) until completion of tissue harvesting, then manually minced for about 5 minutes into a slurry. The tissue slurry was incubated in muscle dissociation buffer (wash media + 1000 U/mL collagenase II [Worthington Biochemical]) at 37°C with 70 rpm agitation for 1 hour. The cells were then washed with wash media and centrifuged at 500*g* for 5 minutes at 4°C. The cells were resuspended in fibroblast growth media (Ham’s F12 media with 10% FBS [Gibco] and penicillin-streptomycin [Gibco]) and were added to gelatin-coated tissue culture plates. Cells were allowed to adhere for 3 hours and supernatant was discarded. The cells (passage 0) were allowed to grow until near confluence and passaged into 12-well plates with coverslips at a seeding density of about 75,000 cells/well. Cells were grown in phenol-red-free Ham’s F12 media supplemented with 10% charcoal-stripped FBS [Gibco] and penicillin-streptomycin [Gibco]. For immunocytochemistry staining, plates were first washed with PBS and fixed in 4% paraformaldehyde for 10 minutes at room temperature. Cells were then washed again with PBS and incubated with permeabilization buffer (0.5% Triton-X in PBS) for 5 minutes at room temperature. Coverslips were blocked with 5% BSA for 1–2 hours at room temperature. Primary antibodies (PDGFRα [R&D Systems AF1062] and ERα [MilliporeSigma 06-935]) in 1% BSA were added to cells and incubated overnight at 4°C. After 2 washes with PBS, coverslips were incubated with respective secondary antibodies (Invitrogen A-31573 for anti-PDGFRα and A32814 for anti-ERα) in PBS for 1 hour at room temperature in the dark. Coverslips were washed 3 times with PBS and incubated in 0.5 μg/mL of DAPI for 5 minutes in the dark and washed 3 times after with distilled water. Coverslips were mounted onto slides with antifade mountant (Invitrogen S36937). Images were obtained using Nikon Ti2 Widefield microscope.

### Flow cytometry.

Single-cell suspensions of lower abdominal and quadriceps muscles were obtained as described above. Cell suspensions passed through a 70 μm cell strainer were used for the flow cytometry staining. Cells were first stained with live/dead fixative stains (Invitrogen L34961, L34974) for 30 minutes on ice in the dark. After 1 round of washing, cells were stained with PDGFRα antibody (Invitrogen 11-1401-82) for 30 minutes on ice in the dark. Next, cells were washed and incubated with fixation and permeabilization buffers (Invitrogen 00-5523-00) according to the manufacturer’s instructions. ERα or C4b primary antibody (MilliporeSigma 06-935, Cedarlane Labs CL7504B) was added, and cells were incubated for 30 minutes on ice in the dark. Secondary antibodies (BioLegend 406410, Invitrogen 12-4739-81 and 62-4137-82) were added for 30 minutes on ice before performing the flow cytometry to validate HAF clusters. For the cell cycle analysis, 1 μL of FxCycle Violet stain (Invitrogen, F10347) was added prior to running flow cytometry. Unstained, single-color controls (SCCs) and fluorescence minus one (FMO) controls were run for each replication. Cells were run on the BD LSRFortessa SORP 6-laser Cell Analyzer or the BD FACSAria SORP Cell Sorter 6-laser. Analysis was performed using FlowJo software. Briefly, single cells were separated out using forward and side scatterplots. Gating for other parameters was set based on SCCs and FMO controls.

### Data availability.

All data generated or analyzed during this study are included in this published article, in the data repositories listed in the references, or in the National Center for Biotechnology Information Gene Expression Omnibus repository (GSE174594).

### Statistics.

Initial analysis of scRNA-Seq data was performed as described above. For other comparisons, the data were first checked for normality using Shapiro-Wilk test and Q-Q plots. For data that did not satisfy the conditions, nonparametric tests were performed. For comparing cell type numbers, a χ^2^ test of proportions was used (101). Post hoc multiple pairwise comparisons were performed via Wilcoxon’s signed-rank test and corrected for multiple comparisons using the Dunn-Bonferroni method (102). For Western blot densitometry comparisons, 2-tailed *t* tests were performed. A *P* value less than 0.05 was considered significant.

### Study approval.

Animal experimental protocols were approved by the Institutional Animal Care and Use Committee at Northwestern University.

## Author contributions

TP, MJT, and HZ performed the experiments. TP performed the computations and analysis of the data. TP and MJT prepared the manuscript with input from all authors. RLL and JJS contributed to the final versions of the manuscript and helped analyze the experiments. SEB and HZ conceived the original idea and supervised the project.

## Supplementary Material

Supplemental data

## Version 1 04/19/2022

In-Press Preview

## Version 2 05/09/2022

Electronic publication
