# Supplementary material for: An estrogen-sensitive fibroblast population drives abdominal muscle fibrosis in an inguinal hernia mouse model
Source: JCI Insight. 2022 Apr 19;7(9):e152011. doi: 10.1172/jci.insight.152011 (PMC9090253; doi:10.1172/jci.insight.152011)
Supplement: Supplemental data [file jciinsight-7-152011-s090.pdf]

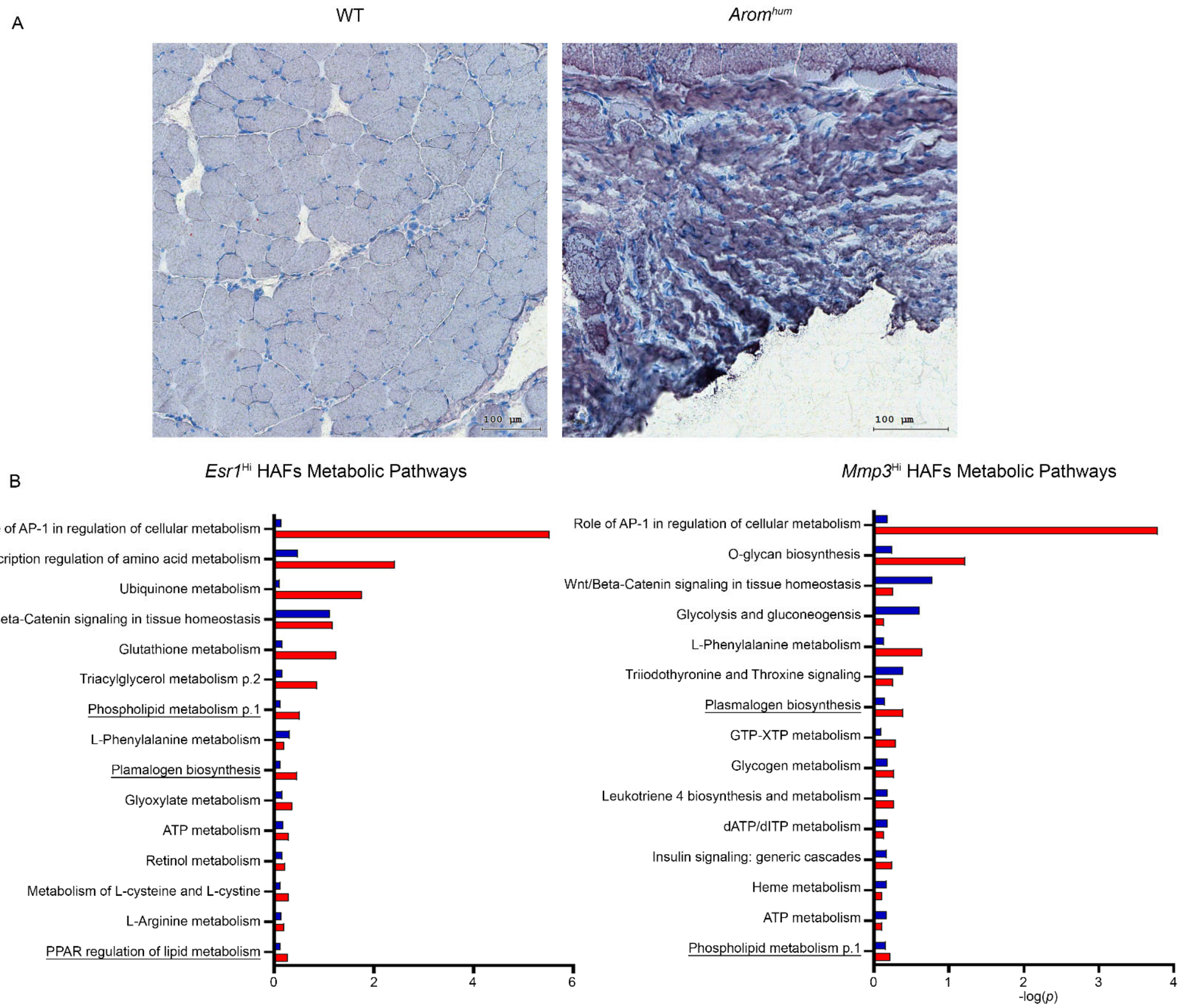

Supplemental Figure 1: (A) Oil Red O staining of WT and *Arom*^{hum} LAM. No apparent lipid droplet accumulation was observed in either tissues. (B) Enriched metabolic pathways in *Esr1*^{Hi} and *Mmp3*^{Hi} clusters with lipid metabolism are underlined. No significant differences were observed between WT and *Arom*^{hum} cells.

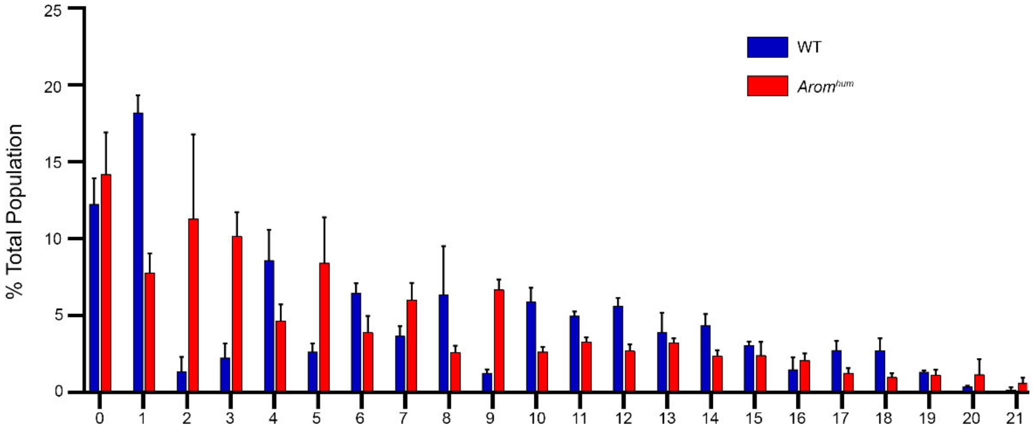

Supplemental Figure 2. WT vs. *Arom*^{hum} cell percentages in each of the 22 cell clusters from Figure 1, D and E.

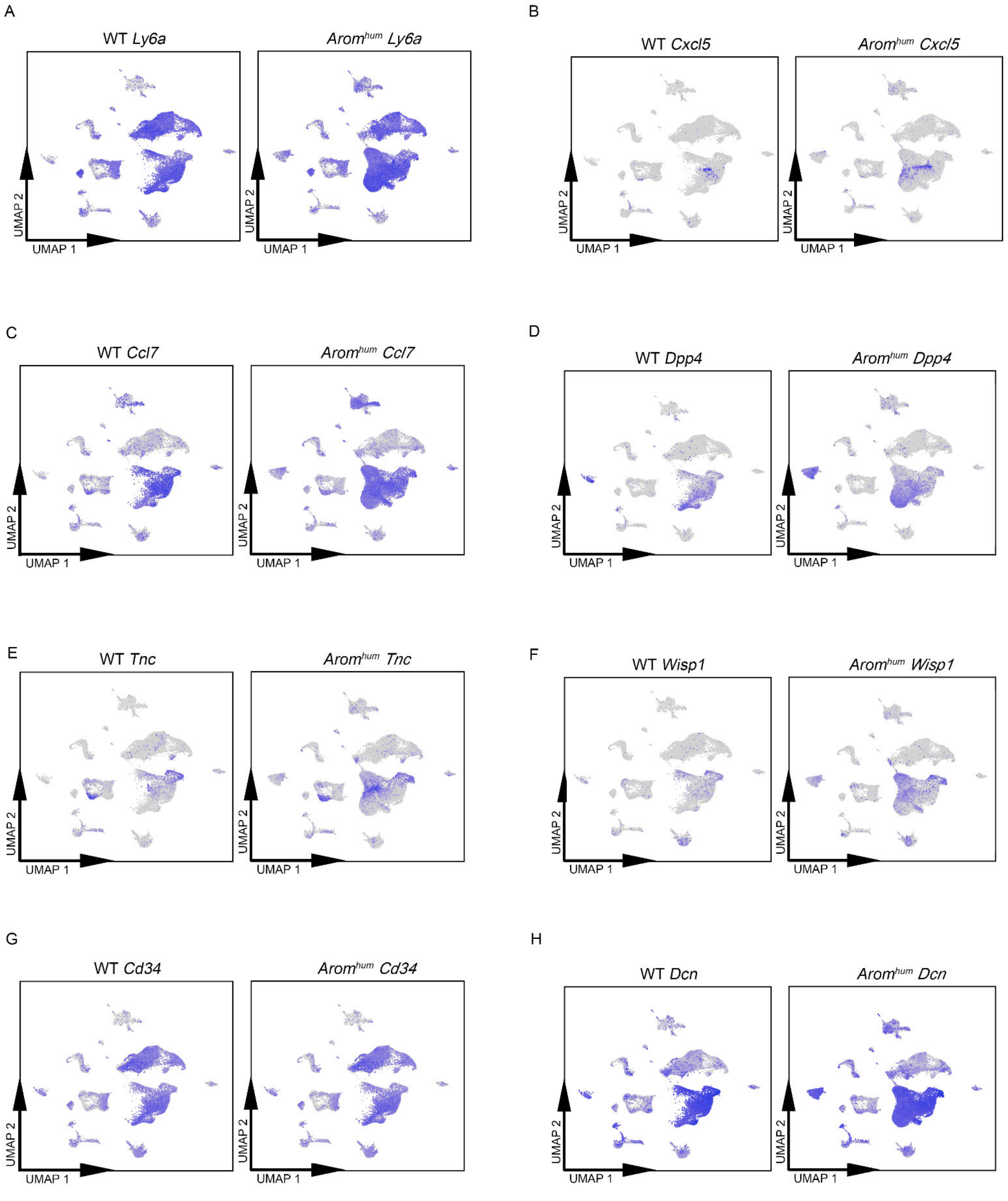

Supplemental Figure 3: Feature plots showing expression of fibro-adipogenic progenitor (FAP) markers (A) *Ly6a* (B) *Cxcl5* (C) *Ccl7* (D) *Dpp4* (E) *Tnc* (F) *Wisp1* (G) *Cd34* (H) *Dcn*

Fibroblast-like Cells Cluster Markers					
**Cluster 0**	**Cluster 2**	**Cluster 3**	**Cluster 6**	**Cluster 15**	**Cluster 16**
**Fibroblast-like Cells**	***Mmp3*** ^{Hi} **HAFs**	***Esr1*** ^{Hi} **HAFs**	**Fibroblast-like Cells**	**Tenocytes**	***Dmkn*** ^{Hi} **Fibroblasts**
Tppp3	Mmp3	Pnoc	Gas1	Fmod	Dmkn
Ccl11	C4b	Ren1	Tgfbi	Tnmd	Sbsn
Abca8a	Cpxm1	Nppc	Angptl1	Comp	Krtdap
Hmcn2	Plac8	Moxd1	Itgbl1	Thbs4	Efemp1
Myoc	Srgn	Emb	Aspn	Abi3bp	Anxa3
Lama2	Cthrc1	Erfe	Fxyd6	Cpxm2	Sema3c
Smoc2	Il33	Chrdl2	Mgp	Fibin	Cd55
Col5a3	Cxcl14	Ramp3	Ramp1	Tgfbi	Aldh1a3
Itih5	Ptx3	Ctla2a	Myoc	Col11a1	Pla1a
Ifi207	Wisp2	Spon2	Prelp	Col12a1	Pcolce2
Crip2	Ccl8	Mdk	Bmp1	Angptl1	Efhd1
Socs3	Sbsn	Mmp19	Pdgfrl	Tnc	Adamts5
Col15a1	Prss23	Crlf1	Prg4	Clec11a	Prss23
Nav1	Cxcl12	Esr1	Plpp1	Fxyd6	Scara5
Icam1	Ptgs2	Ammecr1	C3	Itgbl1	Timp3
Sash1	Procr	Cpe	Fbln1	Fbln7	Ildr2
Ace	Enc1	Rbp1	Smoc2	Ecm2	Dpp4
Podn	Efemp1	Col12a1	Nfe2l2	Col16a1	Wnt2
Osmr	Sfrp2	Tpm2	Gem	S100a4	Opcml
Hsd11b1	Ctla2a	Greb1	Ecm2	Ssc5d	Cmah
Itm2a	Fst	Igfbp5	Lysmd2	Pamr1	Mustn1
Maged2	Nfkbiz	Pgr	Nfib	Srpx2	Qpct
Serpine2	Sfrp1	Socs2	Gas6	Fn1	Igfbp5
Cygb	Ugcg	Tmem100	Tm4sf1	Angptl2	Emilin2
Klf2	Postn	Tmem119	Podn	Plpp1	Dact1
Ramp2	Il1r1	Htra4	Fgl2	Thbs2	Pcsk6
Zeb2	Il6	Plod2	Srpx	Lhfp	Gas7
Ifi211	Nr4a3	Hist1h2bc	Cilp	Cnn3	Basp1
Pdgfrl	Lgmn	Tlnrd1	Serpina3n	Lox	Slco3a1
Mfge8	Prrx1	Maf	Ndrg1	Gas1	Lrrn4cl
Tm4sf1	Col1a1	Fbxo32	Prdx5	Cdkn1c	Il33
Cryab	Col1a2	Rgs10	Cygb	Runx1	Scara3
Pi16	Col3a1	Id2	Vcan	Gng11	Heg1
Crispld2	Cpxm1	Fbln7	Ctgf	Mfge8	Procr
Serpina3n	Adamts5	Cilp	Apod	Tm4sf1	Adgrd1
Gng12	Pla1a	Rbp4	Eln	Mfap4	Cadm3
Lbp	Basp1	Tpm1	Mfap4	Pde4b	Srgn
Fxyd5	Flrt2	Csrp1	Zfp36	Rnf19b	Pde8a
Cyr61	Cd55	Spon1	Wsb1	Wwtr1	Gngt2
Abi3bp	Slfn5	Ncam1	Col14a1	Prg4	Gnpnat1

Supplemental Table 1. Top 50 differentially expressed genes in each of the six fibroblast-like cell clusters. Clusters are labeled with their numbers and their assigned names as in Figure 4C.

Supplemental Methods:

H&E and Masson's Trichrome Staining: LAM tissues from WT and *Arom*^{hum} mice were dissected, fixed in 4% phosphate-buffered paraformaldehyde, embedded in paraffin, and sectioned at 4μm thickness. The sections were stained with hematoxylin and eosin (H&E) stain to observe cellular morphology. Sections were also stained with Masson's Trichrome staining (i.e., Weigert's Hematoxylin, Biebrich scarlet-acid fuschin solution, and Aniline blue) using a staining kit (American Master Tech, # KTTRBPT). Images were obtained using a Zeiss Axio Scope microscope (Zeiss) at ×20 magnification (1).

Western Blotting: Whole LAM tissues were lysed using a Dounce Homogenizer (Active Motif #40401) on ice in a buffer containing 10mM Tris, 150mM NaCl, 1mM EDTA, 0.5% Nonidet P40 and protease inhibitors (Sigma-Aldrich #1187358001, Thermo Fisher Scientific #78441). Lysates were centrifuged for 20 minutes at 4°C at 15,000rpm. BCA assay (Thermo Fisher Scientific #23227) was performed to determine protein concentrations. 40μg of protein was added to the LDS sample buffer (Invitrogen #NP0007) and incubated according to manufacturer's instructions. Samples were separated on 10% SDS-polyacrylamide gels (Thermo Fisher Scientific #NP0324) and then wet transferred to nitrocellulose membranes. The membranes were blocked in 1X fluorescent blocking buffer (Thermo Fisher Scientific #37565) for 30 minutes at room temperature and then incubated with primary antibodies overnight at 4°C (MMP3, Biorbyt, #orb650223; PCNA, Cell Signaling Technologies, #2586s; and TGFBI, Thermo Fisher Scientific, #PA547346). After three washes with Tris-buffered saline with Tween-20 (TBST), membranes were incubated with respective secondary antibodies (Abcam #ab150073, Invitrogen #A32727 and #A21448) in dark for 1 hour at room temperature. After 3 washes with TBST, membranes were imaged at respective wavelengths using iBright 1500 imager. Membranes were also stained with Ponceau S for quantification of total protein. Band signals were measured using Fiji software by using the analyze gels function.

RNAscope®: Chromogenic *in situ* mRNA detection for *Esr1* transcripts was manually performed using the RNAscope 2.5 HD Detection kit (Cat #. 322300, Brown, Advanced Cell Diagnostics Inc., Newark, California). 5-μm thick FFPE tissue sections were pretreated with heat and protease before hybridization. Slides were processed according to the manufacturer's instructions with some modifications: hydrogen peroxide treatment for 30 minutes, AMP 5 hybridization for 45 minutes, and AMP 6 hybridization for 22.5 minutes. Tissue sections were hybridized with RNAscope target probes for *Esr1* (probe Mm-Esr1 cat# 478201). *Esr1* probes target 1045 base-pair-long region in the *Esr1* transcript variants 1 with 20 target-specific double Z probes targeting region 678-1723 in accession number NM_007956.5. Probes to the *DapB* bacterial gene (probe DapB cat# 310043) and the endogenous mouse *UBC* mRNA (probe Mm-UBC cat# 310771) were used as technical negative and positive controls, respectively, for each run. Positive *Esr1* mRNA expression was demonstrated by brown, punctate staining present within the cytoplasm and/or nucleus.

Additional References:

1. Zhao H, Zhou L, Li L, Coon VJ, Chatterton RT, Brooks DC, et al. Shift from androgen to estrogen action causes abdominal muscle fibrosis, atrophy, and inguinal hernia in a transgenic male mouse model. *Proc Natl Acad Sci U S A.* 2018;115(44):E10427-E36.
